# Effects of rituximab in two patients with dysferlin-deficient muscular dystrophy

**DOI:** 10.1186/1471-2474-11-157

**Published:** 2010-07-11

**Authors:** Alberto Lerario, Filippo Cogiamanian, Chiara Marchesi, Marzia Belicchi, Nereo Bresolin, Laura Porretti, Yvan Torrente

**Affiliations:** 1 Department of Neurological Sciences, Fondazione IRCCS Ospedale Maggiore Policlinico, Centro Dino Ferrari, University of Milan, via F. Sforza 35, 20122 Milan, Italy; 2 Centro Interdipartimentale di Citometria, Department of Regenerative Medicine, Fondazione IRCCS Ospedale Maggiore Policlinico, via F. Sforza 35, 20122 Milan, Italy

## Abstract

**Background:**

The administration of rituximab (RTX) *in vivo *results in B-cell depletion, but evidence for multiple mechanisms of action have been reported. Surprisingly, B cell depletion produced a response in patients with polymyositis, which is characterized as a T cell-mediated autoimmune disorder with biopsy findings similar to Miyoshi myopathy (MM). Indeed, in dysferlinopathies, there is evidence of immune system involvement including the presence of muscle inflammation and a down regulation of the complement inhibitory factor, CD55.

**Methods:**

Two patients were treated with four weekly infusions of RTX 375 mg/m2. To measure the improvement in muscle strength after treatment, the isometric hand grip maximal voluntary contraction (MVC) was measured by load cell four times during treatment, and again after one year. In order to assess the reproducibility of our grip assessment, we determined the hand MVC analysis in 16 healthy subjects. Moreover, we measured the number of B cells present in patients by flow cytometric analysis during the course of treatment.

**Results:**

The analysis of B cell number during the course of treatment showed that CD20- and CD19-positive cells were depleted to 0-0.01%. The decrease in B cells was followed by an improvement in the mobility of the pelvic and shoulder girdles as shown by the MRC%. The MVC values of both patients began at values lower than normal whereas during treatment patients had improved percentage of muscle strength. The strength peak in both patients coincided with the minimum B cell values. There were no severe adverse events associated with an infusion of RTX.

**Conclusion:**

We consider the increase in muscle strength observed in both treated patients to be a consequence of their treatment with RTX. To our knowledge, these are the first cases of increased muscle strength in patients with MM. Furthermore, the results of this study indicate that B cell depletion with RTX may be useful in the treatment of patients affected by MM, suggesting a possible role for B cells in the pathophysiology of this muscle disorder.

## Background

Dysferlinopathies are recessive inherited muscular dystrophies caused by mutation of the dysferlin gene (DYSF) mapped to human chromosome 2p13 [1]. The two main phenotypes recognized in such conditions are Miyoshi myopathy (MM), a disorder that preferentially affects the distal musculature, and Limb-Girdle muscular dystrophy type 2B (LGMD2B), a disorder that is characterized by involvement of the pelvic and shoulder girdles [2]. The DYSF gene encodes a 230-kDa protein, which is normally expressed in the sarcolemma in skeletal muscle and which is absent in patients with LGMD2B or MM. Lack of DYSF may cause faulty myoblast fusion, impairing muscle regeneration. Both phenotypes can be found among patients belonging to the same family; thus, they appear to share the same mutation [2]. A biopsy will not detect specific alterations such as variation in fibre size or necrotic and regenerating fibres with increased connective tissue. The specific diagnosis is completed by the use of an immunoblotting technique with anti-DYSF antibodies, which shows an absence of the protein. The immunostaining of sections can also reveal the lack of protein.

In MM, the disease onset generally occurs in the late teens with an initial involvement of the distal muscles in the posterior compartments of the lower limb. A common early symptom is the inability of patients to stand on their tiptoes and to toe walk. Although DYSF is also expressed in cardiomyocytes, there is no evidence of cardiac muscle dysfunction in DYSF-deficient patients. Miyoshi myopathy is associated with markedly elevated creatine kinase levels (10 times normal) and dystrophic changes in muscle histopathology.

The biopsy may also show an inflammatory infiltrate which mimics the histopathologic picture of an inflammatory myopathy [3]. There are several signs that inflammation contributes to dysferlinopathy [4]. Up to now, several studies have reported a prominent inflammatory response in dysferlinopathy patients. In the animal model of DYSF deficiency, i.e. the mutant SJL mouse strain, the disease process was initially considered an 'inflammatory' form of muscular dystrophy. Moreover, a down-regulation of the complement inhibitory factor, CD55 in the skeletal muscle of mice and patients with DYSF-deficient muscular dystrophy was recently demonstrated, resulting in an increased susceptibility of DYSF-deficient human myotubes to complement attack *in vitro *[5]. Since there are no effective therapies to treat MM [6], and the possible therapeutic effects of intravenous immunoglobulins (i.v. Ig) is the inhibition of complement factors C4 and C5, treatment with i.v. Ig was tested in one patient with LGMD2B [7]. After the treatment, the patient experienced a transient improvement in muscle strength in a few muscles [7]. These finding suggest a relationship between the absence of DYSF and immune system abnormalities in muscle, and open the possibility of testing new immunosuppressive treatments in dysferlinopathies. Taking these aspects into consideration, rituximab therapy may be an attractive treatment option for several reasons: muscle infiltrates in dysfelinopathic patients are often difficult to distinguish from those of polymyositis patients, and recent studies reported an improvement in muscle strength without significant side effects in patients with polimyositis treated with rituximab [8]. Moreover, the onset of action of rituximab seems to be relatively rapid and its short-term safety profile is favorable [9]. Experience from the lymphoma literature suggests a low incidence of adverse effects with the medication, which is mostly related to infusion reactions after the first dose [10]. Rituximab is a human/murine chimeric monoclonal antibody, directed against CD20-positive B cells, that has shown promising activity in the treatment of a broad array of autoimmune diseases including rheumatoid arthritis (RA), systemic lupus erythematosus (SLE), idiopathic thrombocytopenic purpura, and IgM-mediated neuropathies [11]. Administration of rituximab *in vivo *results in B-cell depletion, but evidence for multiple mechanisms of action are reported and it remains unclear which mechanisms are most important in patients [11]. In fact, it is somewhat surprising that B cell depletion elicited a striking response in patients with PM, which is characterized as a T cell-mediated autoimmune disorder [8]. We describe two patients with MM in whom the muscle biopsies exhibited an evident myopathic pattern, with necrosis and an increase in interstitial inflammatory infiltrate. After one year of follow-up, the two treated patients did not develop side effects and showed a partial recovery of their distal muscle strength, suggesting that the use of rituximab in these patients is safe and partially active. To our knowledge, this is the first case of increased muscle strength in patients with MM who received an experimental treatment.

## Methods

### Study group and inclusion criteria

A medical and medication history was taken, and a complete physical and neurologic examination and an evaluation of muscle strength by manual testing were conducted. A complete blood cell count, electrolyte levels, creatine phosphokinase (CPK) levels, quantitative immunoglobulins, and CD20 and CD19 levels were recorded. Patients were excluded if they were known to have severe heart disease (heart failure, arrhythmias, coronaropathies, or myocardial infarct in the past 6 months), symptomatic macroglobulinemia, hypersensitivity to any component of rituximab, or pre-existing known malignancy requiring treatment. Patients were also excluded if they had active infections, hepatitis B positivity, hepatitis C positivity, or human immunodeficiency virus positivity. Patients were recruited after obtaining informed consent.

The end point of this study was improvement in muscle strength after treatment with rituximab at the 1-year follow up. The primary efficacy outcome parameter in this study was maximal hand grip strength measured by load cell (BC302; DS Europe, Milan, Italy) [12]. This was then compared with standardized normal levels for age and sex to derive a percentage of normal strength. Treatment was considered to be effective if muscle strength was improved by >5%.

Patient 1 was a 41-year-old man who was diagnosed with MM at 22 years of age. He was born at term following an unremarkable pregnancy. His motor development was normal. There was no family history of muscle diseases. The onset of symptoms began at the age of 16 years when he started to complain of difficulty climbing stairs and standing on his toes. He developed progressive walking difficulties and lost independent ambulation at the age of 30 years. He also developed upper limb involvement. At the time of diagnosis, his right quadriceps muscles showed evidence of a myopathic pattern with necrosis and an increased interstitial cellularity. Immunostaining and western-blotting showed an absence of DYSF protein. A chromosomal analysis showed a homozygous deletion of exon 55 (6233del CCTTCAGC) of the DYSF gene.

In order to determine a baseline for the distal muscle force of the superior arms, the patient's hand grip muscle strength was assessed before (-365, -300, -150, and -90 days) and at times that coincided with the four weekly rituximab infusions (0, 7, 14, 21 days). The same analysis was done 338 days after the first infusion of rituximab.

Patient 2 was a 30-year-old man diagnosed with MM at the age of 17 years. He was born at term following an unremarkable pregnancy. His motor development was normal. A family history of muscle disease was not reported. The onset of symptoms began at the age of 16 years when he started to complain of difficulty standing on his toes and rigidity of the gastocnemii muscles. At the same age, hyperCKemia was observed. From the age of 18 years, the patient developed progressive weakness in the lower limbs, which was more pronounced in the distal compartments, with gait difficulties. In the following years, he also manifested upper limb girdle involvement. At the time of diagnosis, his right quadriceps muscles showed evidence of a myopathic pattern with fiber size variability, increased connective tissue, necrosis, and increased interstitial cellularity. Immunostaining and western-blotting showed an absence of DYSF protein. The chromosomal analysis demonstrated a homozygous deletion of exon 22 (20077delC) corresponding to an amino acid substitution, His693ThrfsX4.

In order to define a baseline for the distal muscle force of the superior arms, the patient's hand grip muscle strength was assessed before (-365, -300, -150 and -90 days) and at times that coincided with the four weekly rituximab infusions (0, 7, 21, 35 days). The same analysis was done 223 days after the first infusion of rituximab. In both patients 1 and 2, we assessed the MRC% index before, during, and after the treatment as indicated in the literature [13].

### Administration of rituximab

Patients were required to provide written informed consent. They received four intravenous infusions of rituximab given at weekly intervals. Premedication with acetaminophen and diphenhydramine was given at each infusion to attenuate possible infusion-related reactions.

During each infusion, patients were treated with rituximab 375 mg/m2. In all cases, the first infusion of rituximab was administered intravenously at an initial rate of 50 mg/hour, and in the absence of hypersensitivity or infusion-related events, the infusion rate was increased in 50-mg/hour increments every 30 minutes to a maximum of 400 mg/hour. Subsequent infusions of rituximab were administered at an initial rate of 100 mg/hour and increased in 100-mg/hour increments at 30-minute intervals to a maximum of 400 mg/hour, as tolerated [14].

### Hand grip maximal voluntary contraction (MVC)

**Figure 1:**
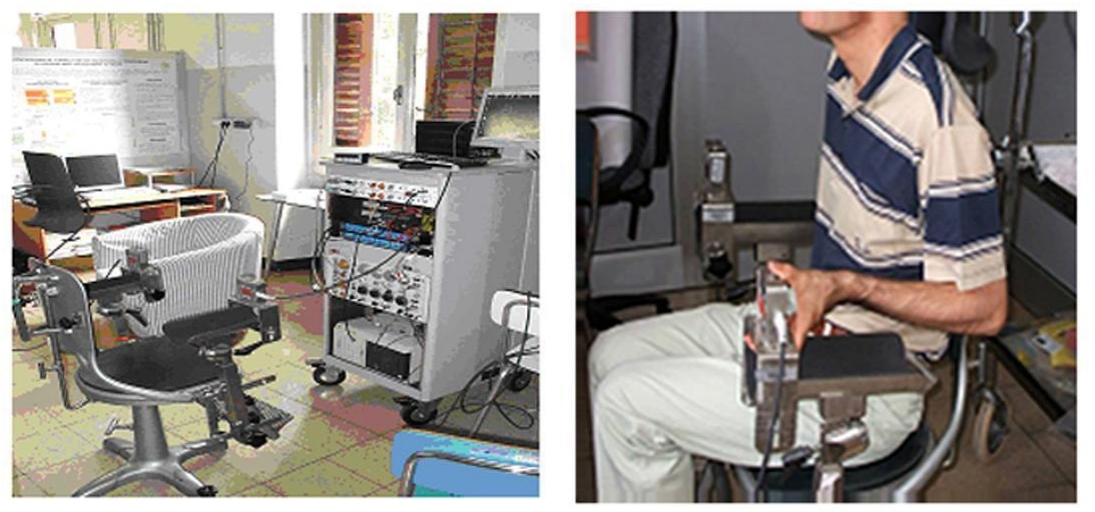
**The load cell**. Illustration of the load cell used for the hand grip muscle strength measurements used in the study.

The maximal voluntary contraction (MVC) isometric, developed from the opponent muscles of the thumb and the flexor muscles of the hand that are involved in hand grip, was measured using one load cell (BC 302; DS Europe, Milan, Italy) [12,15]. The patients were seated in a chair with the left elbow placed on a padded support at a 90° angle. Then they gripped the load cell (BC302; DS Europe, Milan, Italy) between the phalanx of the first (I) finger and the phalanxes of the II, III, IV, and V fingers (Figure 1). The motor task required the subject to exert a hand grip that activated the opponent muscles of the thumb and the flexor muscles of the hand. To determine the MVC force, patients were instructed to increase the force from zero to maximum and to hold it for 3 minutes. Patients performed ten MVC trials subdivided into two sessions and rested for 60 minutes between successive trials. The mean of the best five performances was taken as the MVC force and used as the reference value to calculate the target force. To estimate intra-subject reproducibility of the measured isometric MVC, the MVC was determined twice over a period of 8 days in 16 healthy subjects using the same procedure. The signal from the load cell (range: 0-60 kg; sensitivity, 2 mV/V; total error, < 0.5%; repeatability, < 0.1%) was preamplified and low-pass filtered (< 1000 Hz) with an analogue amplifier [Signal Conditioner Cambridge 1902; Cambridge Electronic Design (CED), Cambridge, England]). The output signal was digitized (Cambridge Micro 1402; CED) with a sampling rate of 2500 Hz and 12-bit quantization with a 5 V range. The digitized signal was stored on a personal computer and displayed using Spike2 software (version 5.11; CED) [12].

## Results

### Patients

Data from 2 patients, both males aged between 30 and 40 years, were obtained. Both patients had a muscle disorder of long duration (15-20 years), and both were symptomatic with reduced muscle strength in the distal muscles of the lower limbs and above. Before treatment, a neurological examination of patient 1 showed an upper-limb, MRC grade 2/3 muscle strength at the girdle muscles and grade 1/2 distally. The proximal lower extremities were more severely impaired: strength was 1/2 in the ileopsoas, and 1 in the quadriceps femoris and tibialis anterior. After the treatment, patient 1 showed an upper-limb, MRC grade 3 muscle strength in the girdle muscles, and grade 2 distally. The proximal lower extremities strength was 2 in the ileopsoas and 1.5 in the quadriceps femoris and tibialis anterior. Before treatment, patient 2's neurological examination showed an upper-limb MRC grade-3 muscle strength in the girdle muscles and grade 2/3 distally. The proximal lower extremities were more severely impaired: his strength was 2 in the ileopsoas, 1.5 in the quadriceps femoris, and 1 in the tibialis anterior. Following treatment, patient 2 had an upper-limb MRC grade 4 muscle strength in the girdle muscles and grade 3\2 distally. The proximal lower extremities were 3 in the ileopsoas, 2 in the quadriceps femoris, and 1.5 in the tibialis anterior. Therefore, both patients had an increase in MRC% [15] (4% for patient 1 and 5% for patient 2). Both patients showed constant normal CPK levels following treatment with rituximab. Patients were given weekly rituximab treatments for four weeks then were followed for one year.

### Reproducibility

In order to assess the reproducibility of our grip assessment, we determined the hand MVC analysis in a group of 16 healthy subjects, with two readings taken over an 8-day period. After 8 days, differences between values were not found to be statistically significant (P < 0.05) suggesting that any statistically significant increase in strength in the patients will reflect a real change in muscle strength.

### Muscular strength

**Figure 2:**
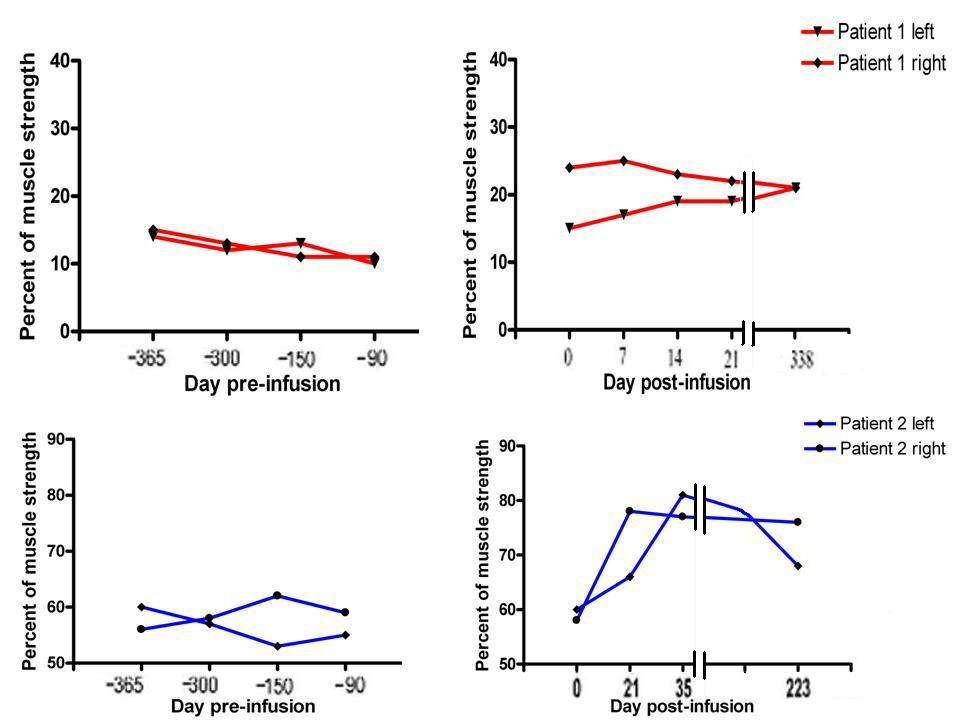
**The time course of changes in the hand-grip muscle strength of patients 1 and 2 before and after treatment with rituximab**. The right and left hand-grip muscle strength is expressed as a percentage of the mean values obtained from a group of 16 healthy male subjects, regarded as baseline, and taken with the value 100%. The upper and lower left panels show the right and left hand grip muscle strength values of patients 1 and 2 at days -365, -300, -150 and -90 before the treatment. Patient 1 was evaluated four times during the course of treatment and again after about one year. The percentage improvement in the patient was around 13% compared to the normal reference value, and the highest percentage increase in strength occurred after 7-14 days. Patient 2 was evaluated three times during the course of treatment and again approximately eight months after treatment. The percentage improvement of the patient compared to the normal reference value was around 58% and the highest percentage increase of distal muscle strength (20%) occurred after 35 days.

**Figure 3:**
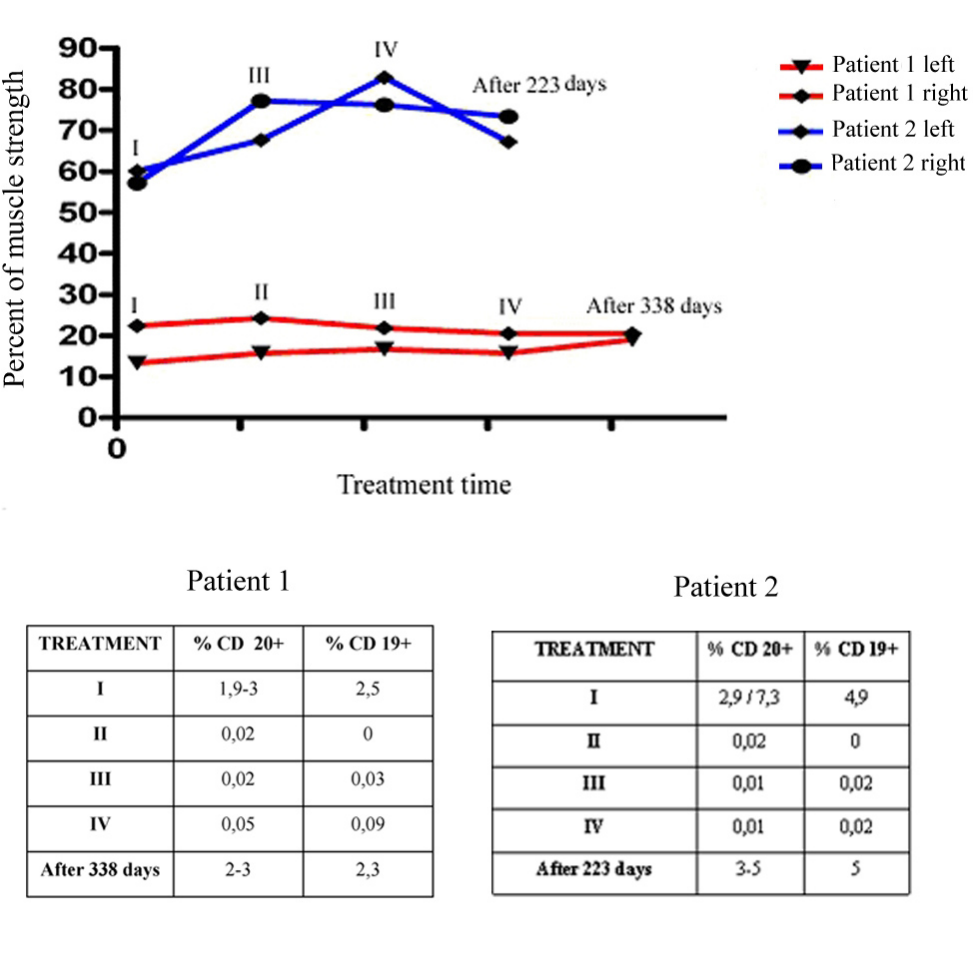
**Correlation between the course of the hand-grip muscle strength and the number of B cells**. The percentages of hand-grip muscle strength of the two treated patients was measured during the four weekly infusions of rituximab (indicated as I, II, III, IV, respectively) and after 338 (for patient 1) and 223 (for patient 2) days of the first infusion of rituximab. The right and left hand grip muscle strength is expressed as a percentage of the mean values obtained from a group of 16 healthy male subjects, which was considered as a value of 100%, and was regarded as the baseline value of the patient. The correlation between the course of the hand-grip muscle strength and the B cells levels show that the depletion of the B cells corresponds to an increase in the hand-grip muscle force, whereas the hand-grip muscle strength returned to the baseline levels as B cell became reconstituted.

The first patient was evaluated four times during the course of treatment and again 338 days after the first infusion. The initial force percentage in both patients, 13% of normal strength for patient 1 and 58% of normal strength for patient 2, was measured -365, -300, -150, and -90 days before the beginning of treatment in four separate hand MVC analysis sessions (Figure 2). Patient 1 was evaluated four times during the course of the study (Figure 2). Maximal improvement in patient 1 occurred seven days after the first infusion, when his muscle strength was 24% of normal, which represented an 11% increase over baseline. Subsequent to the third infusion of rituximab (14 days after the first infusion), the MVC was 21%, and after the fourth infusion (21 days after the first infusion) the MVC was 19%. This muscle percentage strength was retained 338 days after the beginning of treatment. Patient 1's increase in strength was about 10% during the first infusion, suggesting a transient effect of the therapy in the recovery of strength, probably favored by the deletion of B cells. Patient 2 was evaluated three times during the course of treatment and again 223 days after the first rituximab infusion (Figure 2). Maximal improvement occurred after the third infusion (35 days after the first infusion), when the patient's muscle strength was 78% of normal strength, which represented a 20% increase over baseline. Twenty one days after the first infusion, his MVC was 76% and 223 days after the first infusion it was 70%. Both patients were surveyed 21 days from the beginning of treatment and percentage of force was between 15% and 20% compared with healthy in patient 1, while these values were around 72% for patient 2. Thus, the improvement in both patients exceeded the prospectively-defined minimum criterion of effectiveness (>5% increase in muscle strength). An assessment conducted at 338 days for patient 1 and 223 days for patient 2 after treatment with rituximab shows that the percentage of force levels was higher than the values obtained after the first rituximab infusion. A comparison of the values obtained with the hand MVC analysis in both patients indicated that patient 2 had a better response to treatment with rituximab than did patient 1 (Figure 3).

### B cell depletion

The number of B cells measured by flow cytometric analysis during the course of treatment showed that CD20- and CD19-positive cells were depleted to 0-0.01% during the treatment, whereas the percent before treatment was 2.9-7.3% (Figure 3).

Following rituximab infusion, patient 1 exhibited a total depletion of B cells as measured by flow cytometric analysis of CD19 and CD20 levels. Three hundred thirty eight days after the first infusion for patient 1 and 223 days after the first infusion for patient 2, circulating B cells levels returned to normal. In patient 2, B lymphocytes rapidly decreased after the first administration of rituximab and remained nearly undetectable during the entire treatment course. One year after the infusion, the patient's CD19- and CD20-positive cells recovered to the normal range.

### Safety

Rituximab was well tolerated by both patients during treatment and 1 year after completing the treatment. During the first infusion, patient 2 experienced an episode of edema glottis. After momentary interruption of the infusion of the drug, the event resolved spontaneously in 30 minutes. The laboratory parameters were normal.

## Discussion

Currently, there are no effective therapies to treat MM [3]. The use of corticosteroids, particularly dantrolene [6], has led to a slight increase in strength with relative decreases in the levels of CPK. Unfortunately, the many side effects associated with therapy outweigh the modest benefits. The small sample number of patients tested with rituximab therapy must be considered when assessing the results of our study. Indeed, considering the epidemiology of dysferlinopathies (approximately 1/400,000) [2], the preference was to proceed with a pilot study in which each patient was considered his or her own control. In this way, the activity of the drug (strength and concentration of B lymphocytes) over a one-year period was assessed in order to control for any placebo effects.

Rituximab is a genetically engineered (chimeric murine/human) monoclonal antibody directed against the CD20 antigen on B cells [16]. The CD20 surface antigen is widely expressed during B cell ontogeny but is not found on hemopoietic stem cells, pro-B cells, mature plasma cells, or other normal tissues [17-19]. CD20 regulates early steps in activation and differentiation, and may function as a calcium ion channel [20,21]. Rituximab's property of depleting CD20-positive B cells while sparing stem and plasma cells has been used successfully in the treatment of non-Hodgkin's lymphoma (NHL) [22] and in autoimmune disorders such as RA, SLE, and IgM-mediated neuropathies [23-27].

Our one-year study indicates that B cell depletion coincides with improvements in muscle strength in MM patients. Despite the depression of circulating B cell levels, no prominent infections occurred. The results support the notion that B cells play a pivotal role in the pathophysiology of MM. In addition, the duration of B cell depletion and associated reduction in symptoms following a single course of treatment with rituximab in patients with MM is consistent with that seen in other conditions such as NHL and IgM-mediated neuropathies. Both MM patients started with muscle strength values lower than normal (13% in patient 1 and 58% in patient 2). During treatment, both patients experienced an increase in the percentage of muscle strength compared with baseline values. The peak strength for patient 1 increased to about 24% compared with the normal reference value, and the peak strength for patient 2 increased to 78% compared with the normal reference value. The better clinical and MVC% improvement in patient 2 compared with patient 1, who also had a lower response in percentage terms from patient 2, can probably be explained by the different degrees of muscle degeneration at baseline between these patients. Patient 1 experienced the clinical presentation of muscular dystrophy for a greater number of years and may have consequently developed a higher degree of deterioration of his muscle tissue with less chance for recovery.

The development of muscle strength fluctuates over time by 10-20% and, although the muscle strength was always greater than the baseline level, the overall clinical picture of the patients was almost unchanged. In fact, from both descriptions given by the patients and clinical examinations of several muscle districts, substantial improvements were not detected. The possible reasons for this variability are many. On the one hand, the variability may have been related to the method of quantitative measurement. It would take, before treatment, a greater number of manual strength tests in the patients to consolidate the phenomenon of the motor task that, on the one hand, because of variation of the results as it increases the number of trials and other decreases variability minimizing compensating movements made by the patient. It must also take into account the state of health and the lifestyle of the patient when the tests were performed, since the standardization of quantitative evidence of strength is important. The MVC% increases coincided with increases in the MRC% [13] of 4% for patient 1 and 5% for patient 2. The largest increase in the MRC assessment of a single muscle was detected on the distal muscles of the upper limbs. The natural history of MM is characterized by a progressive loss of muscle force with no natural or temporary recovery, as reported in the literature. Thus, the increase in the hand-grip muscle strength observed in both patients and the improvement in the global MRC% can be considered a consequence of the treatment with rituximab. The good tolerability of rituximab in these two MM patients is in accordance with the experience in RA, SLE, and DM [13,28,29] in that there were no severe adverse events associated with the treatment. Such reactions emerge less frequently in patients with autoimmune conditions compared with patients with NHL [27]. In NHL patients, lysis of tumor burden may contribute to the mostly mild-to-moderate transient infusion reactions seen in these patients, the frequency of which is greater during the first infusion than in subsequent infusions [21,30].

The results of this study indicate that B cell depletion with rituximab appears to be useful in the treatment of patients with MM, and suggest a possible role of B cells in the pathophysiology of this muscle disorder. Moreover, the absence of side effects in these patients shows that this treatment is safe. Further investigation is needed to understand the role of B cell depletion in the muscle pathology of MM patients and to determine the effectiveness of rituximab as a treatment for MM.

## Conclusion

The natural history of MM is characterized by a progressive loss of muscle force with no natural, even temporary, recovery. Thus, we can consider the increase in hand grip muscle strength observed in both treated patients and the improvement of the global MRC% as a consequence of the treatment with rituximab. The results of this study indicate that B cell depletion with rituximab appears to be active in the treatment of MM patients, and suggests a possible role of B cells in the pathophysiology of this muscle disorder. Moreover, the absence of side effects during the course of this study demonstrates that this treatment is safe. Further investigations are needed to understand the role of B depletion in the muscle pathology of MM patients and to determine the effectiveness of rituximab as treatment for MM. To our knowledge, this is the first treatment showing increased muscle strength in patients with MM.

## Competing interests

Sources of support: This study was not funded by outside sources, and was a project undertaken by Alberto Lerario and Yvan Torrente at Department of Neurological Sciences, Fondazione IRCCS Ospedale Maggiore Policlinico of Milan. The authors declare that they have no competing interests.

## Authors' contributions

YT conceived of the study, participated in its design and coordination, and helped draft the manuscript. AL was the principal author of the manuscript, and carried out the data collection and statistical analysis. MB and LP carried out the molecular and cellular studies. FG helped with the sampling and data collection process. CM and NB aided in the drafting of the manuscript. All authors read and approved the final manuscript

## Pre-publication history

The pre-publication history for this paper can be accessed here:

http://www.biomedcentral.com/1471-2474/11/157/prepub
